# Immune Thrombocytopenic Purpura Following COVID-19 Infection: A Case Report and Literature Review

**DOI:** 10.7759/cureus.39342

**Published:** 2023-05-22

**Authors:** Hien Nguyen, Michelle Nguyen, Angela Olenik

**Affiliations:** 1 Department of Internal Medicine, Kaiser Permanente, Temple Hills, USA; 2 Department of Pharmacy, Kaiser Permanente, Temple Hills, USA

**Keywords:** itp management, secondary itp workup, vaccine science and policy, covid induced, vaccine-induced thrombocytopenia

## Abstract

Immune thrombocytopenic purpura (ITP) has been described following both coronavirus disease 2019 (COVID-19) infection and COVID-19 vaccination. ITP is a challenging diagnosis of exclusion, and the pathophysiology of these complications is not well understood but believed to be autoimmune in nature. We describe a severe case of ITP following COVID-19 infection in a patient without a history of hematologic or autoimmune disease and his subsequent uneventful course following COVID-19 vaccination. The current Centers for Disease Control and Prevention (CDC) advisory does not identify a history of ITP as a contraindication to COVID-19 vaccination. We compare our study, which describes an uneventful COVID-19 vaccination course with cases that have described recurrences and relapses of ITP following both COVID-19 infection and COVID-19 vaccination. These reports suggest that the placement of some patients into a unique subset among all patients with ITP may be prudent with regard to future COVID-19 vaccination. Through a literature review, we discuss a broader picture of how COVID-19 infection-associated ITP may differ from COVID-19 mRNA vaccination-associated ITP in its demographics, etiology, and outcomes.

## Introduction

After cases of hypercoagulability were described early on in the coronavirus disease 2019 (COVID-19) pandemic in severe cases of infection, a wide spectrum of cases of thrombocytopenia, from mild to severe with life-threatening bleeding, were subsequently reported following infection as well [1-3]. Although mild thrombocytopenia is often diagnosed in patients hospitalized with COVID-19 infection, severe thrombocytopenia with life-threatening clinical bleeding manifestations is much more rarely described [1,3]. We present an elderly male patient who presented with severe thrombocytopenia and bleeding two weeks following COVID-19 infection. We compare and contrast the interesting similarities and differences between COVID-19-induced thrombocytopenia with COVID-19 vaccination-induced thrombocytopenia [1-4]. 

## Case presentation

A 70-year-old male with a past medical history of hypertension, hyperlipidemia, and gout presented with a three-day history of spontaneous bruising over his upper chest, trunk, and arms and red streaks in his saliva. Two weeks prior, he was diagnosed with mild symptomatic COVID-19 infection, when he presented with cough, fatigue, and body aches. He was prescribed Paxlovid but did not end up taking it, as he filled it belatedly past the therapeutic window. He recovered fully following the COVID-19 diagnosis. At this time of presentation, he denied hematochezia, hematuria, or hematemesis, as well as any recent trauma, new medications, or other illnesses. His past medical history was negative for autoimmune or hematologic disorders. The patient’s medications included metoprolol, atorvastatin, and allopurinol. His social history was negative for smoking, alcohol, or illicit drug use. His family history was non-contributory. The patient was previously immunized with the primary COVID-19 Moderna mRNA vaccine series and a single booster dose one year prior. The patient did not experience any adverse reactions, including thrombocytopenia, from these vaccinations.

**Figure 1:**
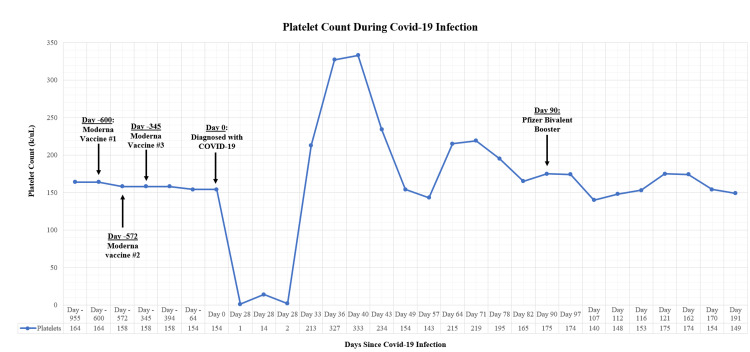
Platelet counts prior to and following COVID-19 Infection and COVID-19 vaccination Reference ranges: 130-400 platelets 10^3^/μL

His vital signs on presentation were a temperature of 97.9F, blood pressure of 120/79 mmHg, respiratory rate of 14 breaths per minute, and heart rate of 80 beats per minute with normal oxygen saturation on ambient air. His vital signs did not reveal orthostatic hypotension, and his mentation was normal. His other physical examination findings were normal except for the skin examination, which showed scattered petechiae located mainly in the upper chest and upper extremities. Laboratory bloodwork revealed a platelet count of 1 x 10^3^ /μL. The lactate dehydrogenase (LDH) level was slightly elevated, and he had normal haptoglobin, vitamin B12 levels, folate levels, and viral hepatitis and HIV panels. The COVID-19 polymerase chain reaction test was negative. The patient was started on dexamethasone 40 mg intravenously and received a transfusion of one unit of platelets. The platelet count increased transiently up to 14 x 10^3^/μL but then fell back down to 2 x 10^3^/μL. He was subsequently treated with two 1 g/kg doses of intravenous immunoglobulin (IVIG), resulting in a recovery of platelet count to 89 x 10^3^ /μL by the date of hospital discharge. His platelet counts at his outpatient follow-up visits remained normal and ranged between 243 and 333 10^3^/μL (reference ranges of 130-400 10^3^ /μL). Three months following the diagnosis of COVID-19 infection, the patient successfully received the bivalent Pfizer mRNA vaccination and maintained stable platelet counts for six months post-vaccination (Figure 1). Therefore, the patient received mRNA vaccinations prior to COVID-19 infection and following infection without the occurrence of thrombocytopenia or other adverse reactions.

## Discussion

Immune thrombocytopenic purpura (ITP) is a poorly understood medical condition that may develop primarily or secondarily to autoimmune processes, viral infections, immunodeficiency, malignancies, or medications [1-3]. ITP has been described as a complication of both COVID-19 infection and COVID-19 mRNA vaccination [3]. Other vaccinations that have been associated with ITP include vaccines for influenza, hepatitis A and B, diphtheria-tetanus-pertussis, and varicella. ITP is a diagnosis of exclusion in a differential diagnosis that also includes especially other life-threatening causes of thrombocytopenia such as thrombotic thrombocytopenic purpura (TTP) and hemolytic uremic syndrome [4]. ITP may present along a spectrum from asymptomatic to mild thrombocytopenia to serious gastrointestinal or intracranial hemorrhage with severe thrombocytopenia. ITP is believed to develop from IgG autoantibodies binding to platelet surface antigens, leading to platelet clearance by splenic macrophages [2,3]. The development of ITP following COVID-19 infection and COVID-19 vaccination is believed to involve autoimmune mechanisms such as molecular mimicry, epitope spreading, and polyclonal activation [1-3].

Most COVID-19 infection-associated ITP cases have been diagnosed during the acute infection. In a systematic review of 55 patients with COVID-19 infection and ITP, 54.8% were male, the mean age was 60 years, and the mean time period from a COVID-19 diagnosis to an ITP diagnosis was 18 days (ranging from same-day diagnosis to 125 days) [5]. The mean time to recovery from ITP was six days (ranging from 2 to 22 days) [5]. These patients had a mean platelet count of 14.5 x 10^3^/μL, and in most of these cases, the remission of ITP occurred with IVIG but not with dexamethasone and platelet transfusion alone. Moreover, in another systematic review of 45 patients with COVID-19 infection and ITP, 7% of these patients did not present with COVID-19 symptoms [6]. Based on these findings, these authors recommend that patients diagnosed with ITP should be tested for COVID-19 infection [6]. In this study, there was a higher rate of thrombocytopenia in patients with severe disease (57.7%)* versus* non-severe disease (31.6%) [6].

In comparison to COVID-19 infection-associated ITP, COVID-19 mRNA vaccination-associated ITP appears to have slightly different demographics - more females *versus* males and at younger ages (50 *versus* 60). To determine the incidence of ITP reported following COVID-19 vaccination, an adverse event search for “immune thrombocytopenia” and “immune thrombocytopenia purpura” was performed in the Vaccine Adverse Event Reporting System (VAERS) database (Table 1) [7]. As of January 29, 2023, a total of 426 unique records were identified. Across three COVID-19 vaccine manufacturers, most cases of ITP were seen in older adults (ages 50 or older) and more in females. In the review of patients with ITP associated with COVID-19 vaccination, as reported in VAERS, the mean platelet count was 12 x 10^3^/μL [7]. This platelet nadir is slightly lower than the counts seen with COVID-19 infection-associated ITP. Like COVID-19 infection-associated ITP, most reported cases of vaccine-induced ITP in the literature were mild, with rare cases of severe ITP responsive to IVIG. There are case reports of ITP following Pfizer-BioNTech, Moderna, and Janssen vaccinations.

**Table 1:** Incidence of ITP post COVID-19 vaccination reported in the United States by vaccine type Data through 01/20/2023 for complete entries were summarized from the Vaccine Adverse Event Reporting System (VAERS). Patients with no reported platelet counts were removed from the total sample when calculating overall percentages. ITP: immune thrombocytopenic purpura

	Vaccine Type			Total (n=426)
	Pfizer/BioNTech (n=207)	Moderna (n=173)	Janssen (n=46)	
Age				
<50 years old	66 (31.9%)	54 (31.2%)	13 (28.3%)	133 (31.2%)
>50 years old	141 (68.1%)	119 (68.8%)	33 (71.7%)	293 (68.8%)
Gender				
Male	83 (40.1%)	67 (38.7%)	20 (43.5%)	170 (39.9%)
Female	116 (56.0%)	103 (59.5%)	26 (56.5%)	245 (57.5%)
Unknown	8 (3.9%)	3 (1.7%)	0 (0)	11 (2.6%)
Vaccine Dose Prior to Presentation				
1	79 (38.2%)	84 (48.6%)	29 (63.0%)	192 (45.0%)
2	58 (28.0%)	48 (27.7%)	0 (0)	106 (24.9%)
>2	23 (11.1%)	12 (6.9%)	0 (0)	35 (8.2%)
Unknown	47 (22.7%)	29 (16.8%)	17 (37.0%)	93 (21.8%)
Platelet Count at Presentation (10^3^/µL)				
Average (Range)	11.5 [<1-108]	13.4 [<1-119]	12.0 [<1-56]	12.3 [<1-119]
<10 x 10^3^/µL*	82 (73.8)	64 (66.7)	16 (88.9)	162 (38.0)
Not Reported	96 (46.4)	76 (43.9)	25 (54.3)	197 (46.2)

The current Centers for Disease Control and Prevention (CDC) advisory states that there are no contraindications to COVID-19 mRNA vaccination in patients with a history of ITP. Various studies support these recommendations. In a recent report, the risk of ITP relapse after receiving the Pfizer-BioNTech vaccine was described as clinically infrequent at a rate of 2.2% after the initial vaccination and 4.3% after the booster dose [7]. Ruzicka et al. reported both long-term and post-subsequent vaccination outcomes in five patients who were diagnosed with *de novo* vaccine-associated ITP. Four of these patients maintained stable platelet counts following up to three additional COVID-19 vaccinations [8]. These authors concluded that re-vaccination of patients who have already experienced ITP following vaccination can be safely performed but recommended that the vaccine be different than the one that triggered the ITP.

Our case describes a patient who experienced severe ITP following COVID-19 infection, but he did not subsequently experience a re-occurrence of ITP following COVID-19 vaccination. However, there are literature reports of unusual, relapsing ITP from both COVID-19 infection and COVID-19 vaccination. Based on these reports, some authors argue that patients with a history of relapsing ITP should be placed into a subset apart from all patients with ITP, with regard to surveillance and COVID-19 vaccination management. Boehm described a patient with COVID-19 who developed *de novo *ITP and then experienced nine relapses of ITP over 10 months [9]. Serrano described two patients whose COVID-19 infection was followed by repeated relapses of severe thrombocytopenia, which required repeated courses of methylprednisolone, followed by rituximab, IVIG, and oral thrombopoietin analogs eltrombopag and mycophenolate [10]. Ratajcza et al. reported a case of a 65-year-old male with a history of ITP, who experienced a relapse of his ITP following the BNT162b2 COVID-19 vaccine [11]. One month following the second dose of this vaccination, he presented with petechiae, asthenia, and thrombocytopenia, which responded to a four-day course of intravenous corticosteroids and intravenous immunoglobulin. His platelet counts remained normal for eight months until he received a booster vaccination after receiving prednisone pre-vaccination. Several days later following this booster, his platelet counts dropped again, and he required prednisone and rituximab therapy [11]. The risk of ITP relapse was predicted by the presence of active ITP, platelet counts under 50, and younger age at the time of vaccination [11]. Such patients may benefit from more surveillance or using a different COVID-19 vaccination than the one that provoked the ITP.

The pathophysiology of COVID-19 infection-induced ITP is believed to involve autoimmune molecular mimicry and hyperstimulation of the immune response. Viral SARS-CoV-2 epitopes may share several homologous peptide sequences with human proteins [2]. Following infection, the immune responses against the SARS-CoV-2 virus may cross-react with self-antigens, leading to autoimmune pathologic sequelae. Hyperstimulation of the immune system by SARS-CoV-2 is a well-documented phenomenon associated with an increase in pro-inflammatory cytokines (cytokine storm) and changes in circulating leukocytes that may also exacerbate this autoimmune process. Autoimmunity triggered by mRNA vaccination-associated ITP is also believed to involve similar mechanisms, including molecular mimicry, epitope spreading, and polyclonal activation. Another mechanism thought to explain vaccine-induced ITP is the presence of adjuvants that are thought to trigger autoimmune/inflammatory syndrome by adjuvants (ASIA), a group of immune-mediated diseases that stem from previous exposure to adjuvants [2]. However, the mRNA Moderna COVID-19 vaccine notably does not contain adjuvants while the mRNA Pfizer COVID-19 vaccine contains an adjuvant that is lipid based [12].

The American Society of Hematology recommends that the main goal of treatment for ITP is to treat or prevent significant bleeding. In patients with critical ITP-associated bleeding, first-line therapies include platelet transfusions, glucocorticoids, and IVIG [1-3]. Dexamethasone 40 mg orally or intravenously once daily for four days is preferred for initial therapy to produce more rapid responses and fewer bleeding events. Alternative glucocorticoids include methylprednisolone or prednisone. IVIG or anti-D therapies are typically reserved for those with contraindications to corticosteroids and/or those requiring a more rapid increase in platelet count. Patients with minor or no signs of bleeding are typically managed with glucocorticoids, IVIG, or anti-D. Second-line agents for patients whose thrombocytopenia is refractory to initial therapies include rituximab and thrombopoietin receptor agonists (romiplostim, eltrombopag, avatrombopag), or rarely, splenectomy [1-3].

## Conclusions

The conclusion remains that the benefits of COVID-19 vaccination exceed the risks of no immunization. But these advantages have been tempered by recent observations that mRNA COVID-19 vaccinations are now showing significantly falling efficacy. As mass vaccination continues to expand globally, increased surveillance and monitoring of adverse events associated with COVID-19 infection and vaccination are needed to better identify and mitigate potentially serious hematological complications such as ITP. In addition, more data should be collected to establish a causal relationship between COVID-19 vaccination and sequential ITP to recommend and formulate guidelines for safe administration in those with a history of pre-existing ITP or *de novo* ITP following COVID-19 infection. Some authors argue that a subset of patients with a prior history of relapsing ITP should be considered unique among all patients with ITP in the management of COVID-19 mRNA vaccination.
